# Colistin-degrading proteases confer collective resistance to microbial communities during polymicrobial infections

**DOI:** 10.1186/s40168-022-01315-x

**Published:** 2022-08-19

**Authors:** Do-Hoon Lee, Ju-Hee Cha, Dae-Wi Kim, Kihyun Lee, Yong-Seok Kim, Hyo-Young Oh, You-Hee Cho, Chang-Jun Cha

**Affiliations:** 1 Department of Systems Biotechnology and Center for Antibiotic Resistome, Chung-Ang University, Anseong, 17456 Republic of Korea; 2 Division of Life Sciences, Jeonbuk National University, Jeonju, 54896 Republic of Korea; 3 Department of Pharmacy, College of Pharmacy and Institute of Pharmaceutical Sciences, CHA University, Seongnam, 13488 Republic of Korea

**Keywords:** Colistin, Antimicrobial resistance, Colistin-degrading protease, Collective resistance, Polymicrobial infection, *Stenotrophomonas maltophilia*

## Abstract

**Background:**

The increasing prevalence of resistance against the last-resort antibiotic colistin is a significant threat to global public health. Here, we discovered a novel colistin resistance mechanism via enzymatic inactivation of the drug and proposed its clinical importance in microbial communities during polymicrobial infections.

**Results:**

A bacterial strain of the Gram-negative opportunistic pathogen *Stenotrophomonas maltophilia* capable of degrading colistin and exhibiting a high-level colistin resistance was isolated from the soil environment. A colistin-degrading protease (Cdp) was identified in this strain, and its contribution to colistin resistance was demonstrated by growth inhibition experiments using knock-out (*Δcdp*) and complemented (*Δcdp::cdp*) mutants. Coculture and coinfection experiments revealed that *S. maltophilia* carrying the *cdp* gene could inactivate colistin and protect otherwise susceptible *Pseudomonas aeruginosa*, which may seriously affect the clinical efficacy of the drug for the treatment of cystic fibrosis patients with polymicrobial infection.

**Conclusions:**

Our results suggest that Cdp should be recognized as a colistin resistance determinant that confers collective resistance at the microbial community level. Our study will provide vital information for successful clinical outcomes during the treatment of complex polymicrobial infections, particularly including *S. maltophilia* and other colistin-susceptible Gram-negative pathogens such as *P. aeruginosa*.

Video abstract

**Supplementary Information:**

The online version contains supplementary material available at 10.1186/s40168-022-01315-x.

## Background

Colistin, also known as polymyxin E, is a lipopeptide antibiotic produced by *Paenibacillus polymyxa* subsp. *colistinus* [1]. Since its discovery in 1949, it was used therapeutically to treat Gram-negative bacterial infections. However, in the early 1980s, its clinical usage was reduced substantially due to adverse effects, such as nephrotoxicity and neurotoxicity, and the advent of newer and less toxic options, such as cephalosporins and carbapenems [2]. In the early 2000s, the increasing prevalence of infections by multidrug-resistant (MDR) bacteria led to the revival of colistin for the treatment of such infections [3]. It is also referred to as a “last-resort antibiotic” because, in certain cases, it is the only effective antibiotic against MDR Gram-negative pathogens [4]. Lipopolysaccharide (LPS) modification, efflux pump, hyperproduction of polysaccharides, and, in rare cases, a complete loss of LPS are known as resistance mechanisms employed against colistin by bacteria [5]. These colistin resistance mechanisms were observed only in bacterial chromosomes, until the plasmid-mediated colistin resistance gene, referred to as mobile colistin resistance (*mcr*) gene encoding a phosphoethanolamine transferase, was identified recently [6]. This gene has been detected worldwide since its emergence, and numerous variants have also been identified [7].

A few decades ago, an enzyme called colistinase that could cleave and inactivate colistin was reported from the colistin-producing *Paenibacillus polymyxa* (formerly named *Bacillus polymyxa*) strain [8, 9]. Recently, a gene responsible for the cleavage was identified to be a serine alkaline protease in *Bacillus licheniformis*, which was proposed as a self-defense system to contribute to protection against antimicrobial peptides produced by the Gram-positive antibiotic producers [10]. However, these proteases have not been clearly demonstrated to be an antibiotic resistance determinants [11]. Furthermore, they could not be considered an urgent threat in the clinic until they are observed in Gram-negative pathogens, because colistin is less active against Gram-positive bacteria and usually administered to patients with bacterial infections caused by MDR Gram-negative pathogens [12]. Therefore, a pre-emptive characterization of the colistin-inactivating resistance mechanism in these pathogens is required prior to the recognition as an emerging resistance in the clinic.

Many bacterial infections including cystic fibrosis (CF) lung infection are caused by polymicrobial communities rather than a single pathogen [13]. This complexity has sometimes led to the failure of antibiotic treatment in the clinic. For example, antibiotic eradication therapy for *Pseudomonas aeruginosa* in CF patients failed in 10–40% of patients [14], which may be caused by variations in the host, pathogen, and polymicrobial interaction [15, 16]. Particularly, polymicrobial interactions in CF patients can significantly influence the efficacy of antibiotic treatment, although the underlying mechanisms remain poorly understood [17, 18]. Recently, Bottery et al. suggested the possible mechanisms of interspecies interaction in polymicrobial infection, thereby leading to collective resistance mediated by the presence of antibiotic-inactivating enzymes within the polymicrobial community [17]. They revealed that the chromosomally encoded metallo-β-lactamase of *Stenotrophomonas maltophilia* which commonly coinfects CF lung provided imipenem exposure protection to otherwise sensitive *P. aeruginosa* by detoxifying the lung environment [18]. Several studies have also emphasized the importance of microbial interaction during polymicrobial infections, indicating that it is critical to evaluate antimicrobial resistance in the context of microbial communities (so-called collective resistance) rather than at a single species level [19–23].

Here, we isolated an environmental strain of *S. maltophilia* that was highly resistant to colistin and capable of cleaving and inactivating the antibiotic. We also characterized the contribution of the colistin-inactivating enzyme to colistin resistance, its ability to provide protection to other coinfecting pathogens in polymicrobial infection communities, and its evolutionary features to evaluate its potential menace in the clinical settings.

## Results

### Cleavage of colistin by *Stenotrophomonas maltophilia* strain Col1

**Fig. 1:**
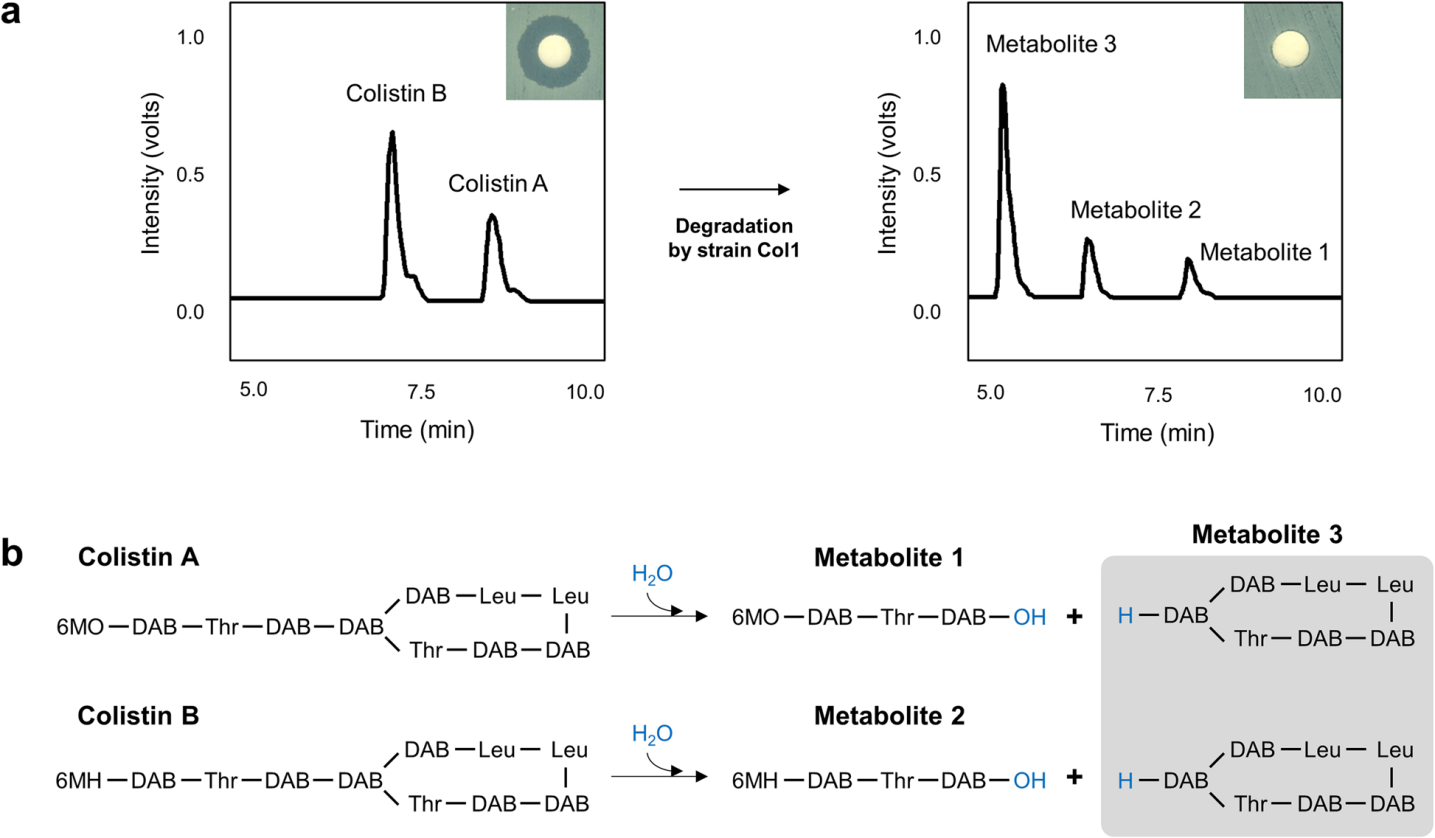
Cleavage and inactivation of colistin by *S. maltophilia* strain Col1. **a** HPLC chromatograms and disk diffusion assay results (inset) from culture supernatants. **b** A proposed mechanism of the cleavage of colistin by *S. maltophilia* strain Col1. DAB, MO, and MH indicate l-diaminobutyric acid, 6-methyloctanoyl, and 6-methylheptanoyl, respectively

*S. maltophilia* strain Col1 exhibiting a high-level resistance against colistin [a minimum inhibitory concentration (MIC) value of 32 mg/L] was isolated from the soil environment. Inactivation of the drug by this strain was analyzed by assessing the antimicrobial activity of colistin remaining in submerged cultures. Disk diffusion assay against the colistin-susceptible *E. coli* DH5α using the culture supernatant of strain Col1 spiked with colistin revealed that strain Col1 could completely inactivate the drug. Three metabolites were detected from the culture supernatant, and the conversion resulted in the loss of antimicrobial activity of colistin (Fig. 1a). The chemical structures of the metabolites were elucidated by LC–MS/MS. The following compounds were identified: 6-methyloctanoyl-l-diaminobutyric acid (DAB)-l-Thr-l-DAB-OH (metabolite 1), 6-methylheptanoyl-l-DAB-l-Thr-l-DAB-OH (metabolite 2), and the cyclic peptide moiety of colistin (metabolite 3) (Fig. 1b and Additional file 1: Figs. S1-S3).

### Identification and characterization of the colistin-degrading enzyme

The colistin-degrading enzyme activity was detected only in the culture supernatant but not in the cell-free extract. The extracellular fraction of the culture of strain Col1 in the stationary growth phase was used to purify the enzyme. The purified protein was identified as a serine protease (CDS No. 00541 of strain Col1 genome) by LC–MS/MS analysis and SEQUEST search. According to the MEROPS database [24], the protein was assigned to a subfamily S08A of subtilisin protease, and the closest hit in the database was S08.110 (keratinase K1 of *S. maltophilia*). Its orthologous proteins were widely distributed in *Xanthomonadaceae.* However, the highly homologous proteins (> 70.7% identity) were only detected in *S. maltophilia*, implying that the protease-coding gene has a unique lineage within *S. maltophilia*. The protease showed a low sequence identity (33.9%) compared to the previously characterized colistin-degrading alkaline protease (Apr) from *Bacillus licheniformis* [10], which belonged to the same subtilisin protease family. The protein was named colistin-degrading protease (Cdp).

### Role of Cdp in colistin resistance

The presence of colistin-inactivating protease in strain Col1 suggests that the enzyme could confer resistance against colistin to this strain. To assess the role of the protease in colistin resistance, knock-out and knock-in mutants were constructed. The full gene was deleted to develop the strain Col2 (*Δcdp*), and the gene was re-introduced to strain Col2 to generate the complemented mutant strain Col3 (*Δcdp::cdp*).

The colistin-degrading activity was monitored for the three strains (Col1 to Col3 strains) throughout their growth. For the wild-type strain (Col1), the colistin-degrading activity was detected only after the transition growth phase, whereas the strain with *cdp* deletion (Col2) showed no activity throughout the growth period, indicating that the protease was solely responsible for the colistin degradation (Additional file 1: Fig. S4). In the case of the strain complemented with the *cdp* gene (Col3), a low level of activity was detected during the early growth phase, and the activity increased rapidly during the exponential growth phase. These results may be attributed to the combined use of its own promoter and an exogenous promoter in the vector system, which allowed earlier expression at a higher level. The initial attempts to differentiate the colistin susceptibility of these strains were not successful because the levels and the timing of Cdp expression could not be consistently used for conventional antimicrobial susceptibility testing (AST) methods.

**Fig. 2:**
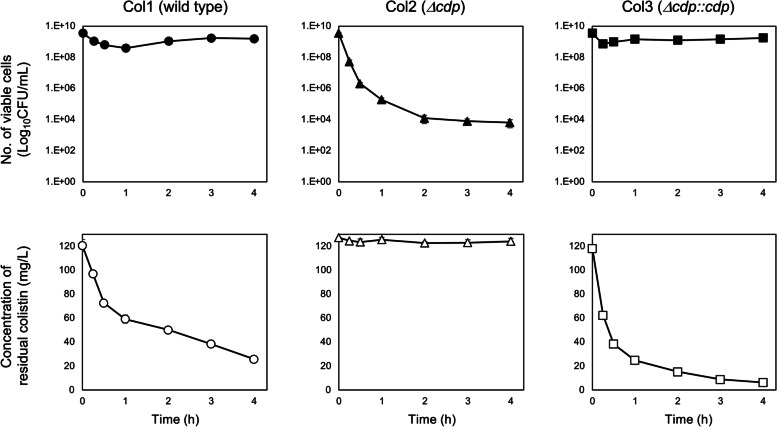
Inhibition of growth by colistin and colistin degradation in the cultures of *S. maltophilia* strains. Colistin was added to 12-h cultures at a concentration of 128 mg/L. The number of viable cells was determined as colony-forming units (CFU). Circle, triangle, and square indicate strains Col1 (wild type), Col2 (*Δcdp*), and Col3 (*Δcdp::cdp*), respectively. Closed and open symbols indicate viable cell count and concentration of residual colistin in the cultures, respectively

To assess the role of Cdp in colistin resistance at the late growth phase, a relatively high concentration of colistin (fourfold higher than the MIC of the wild-type strain Col1) was spiked into the cultures of the strains at the stationary growth phase (12-h cultured). The colistin treatment resulted in the inhibition of the growth of strain Col2, whereas strains Col1 and Col3 could survive due to the degradation of colistin by protease. The survival of these strains coincided with the reduced amount of residual colistin in the culture supernatants (Fig. 2). These results indicated that in the late growth phase, Cdp could contribute to colistin resistance of these strains by inactivating the drug.

### Colistin exposure protection to *P. aeruginosa* provided by Cdp of *S. maltophilia*

*S. maltophilia* is frequently isolated together with other pathogenic bacteria, such as *P. aeruginosa* from cystic fibrosis (CF) patients [25, 26]. *P. aeruginosa* is an opportunistic pathogen responsible for life-threatening acute and chronic infections, and colistin is usually used for the treatment of CF infections caused by MDR *P. aeruginosa* [27]. Here, we examined the colistin exposure protection to the model organism *P. aeruginosa* strain PAO1 provided by *S. maltophilia* Col1 and its mutants. When the culture supernatant of the strain Col1 or Col3 (carrying the *cdp* gene) obtained at the late growth phase was added to the MIC assay medium, the MIC value of the strain PAO1 increased from 2 to 8 mg/L. In contrast, the MIC value remained unchanged when the supernatant of the strain Col2 was added. These results suggest that the presence of colistin-degrading protease could lead to the survival of the coexisting strain.

**Fig. 3:**
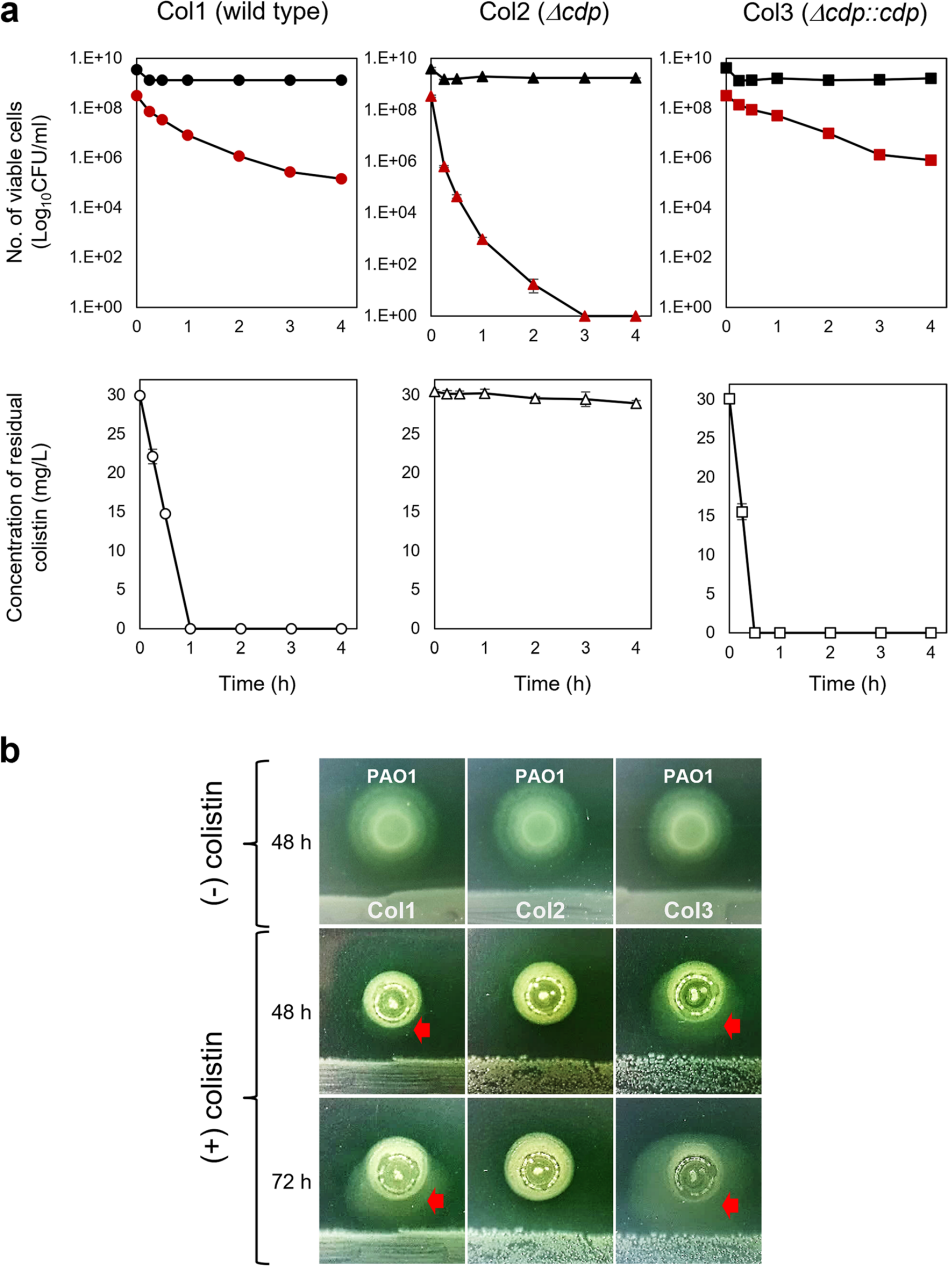
Colistin exposure protection to *P. aeruginosa* provided by Cdp-producing *S. maltophilia* strains during cocultures. **a** Planktonic coculture assay. Changes in viable cell number were estimated for *P. aeruginosa* strain PAO1 (red) and *S. maltophilia* strains (black). The concentration of residual colistin in cocultures was measured after colistin spike (32 mg/L). **b** Solid agar coculture assay. The bacterial lawn of *S. maltophilia* strains was positioned at the bottom. Red arrows indicate the spreading growth of *P. aeruginosa* strain PAO1 toward *S. maltophilia* strains

In addition, we analyzed the changes in viable cell numbers of *P. aeruginosa* when this pathogen was planktonically cocultured with *S. maltophilia* strains in the presence of colistin. Strains Col1 and its mutants showed no significant changes in their viable cell numbers after colistin was spiked. When the strain PAO1 was cocultured with the strain Col2, the cells were almost completely killed within 3 h. However, PAO1 cells survived when cocultured with *cdp*-carrying strains (strains Col1 and Col3) (Fig. 3a). These results were consistent with the observation that colistin rapidly disappeared in cocultures with strains Col1 and Col3, and the concentration of residual colistin remained unchanged in the coculture with strain Col2 (Fig. 3a), indicating that the survival of strain PAO1 was mediated by Cdp-dependent inactivation of colistin. Similar results were also obtained with *A. baumannii* when cocultured with *S. maltophilia* strains (Additional file 1: Fig. S5). These planktonic coculture experiments could mimic coinfection events such as bloodstream infections causing septicemia. Our results corroborate that the presence of the protease-producing *S. maltophilia* strain could lead to the acquisition of colistin resistance within the bacterial community involved in polymicrobial infections.

*P. aeruginosa* is one of the most common bacterial pathogens observed in CF respiratory tract infections [27, 28]. In these chronic infections, *P. aeruginosa* and *S. maltophilia* colonize and form highly populated biofilms rather than undergoing planktonic growth [29, 30]. A solid agar coculture assay was performed to understand the interaction between the two species. Strain PAO1 displayed a spreading growth due to its motility on agar media in the absence of colistin (Fig. 3b). When PAO1 cells (> 10^5^ cells) were inoculated in spots located in proximity to each of *S. maltophilia* strains onto agar media containing colistin with a fivefold higher amount compared to the MIC value of strain PAO1, their spreading growth was retarded at 48 h in cocultures with strain Col1 and Col3, and the growth was not observed in coculture with the strain Col2 devoid of the *cdp* gene (Fig. 3b). Interestingly, the strain PAO1 showed a marked spreading growth at 72 h only when cultured in the proximity of protease-producing *S. maltophilia* strains (Col1 and Col3). Hazy haloes produced with the spreading growth of the strain PAO1 advanced toward the *S. maltophilia* strains (Fig. 3b). The size of the haloes increased in a protease activity-dependent manner.

### Antibacterial efficacy of colistin during bacterial coinfection

**Fig. 4:**
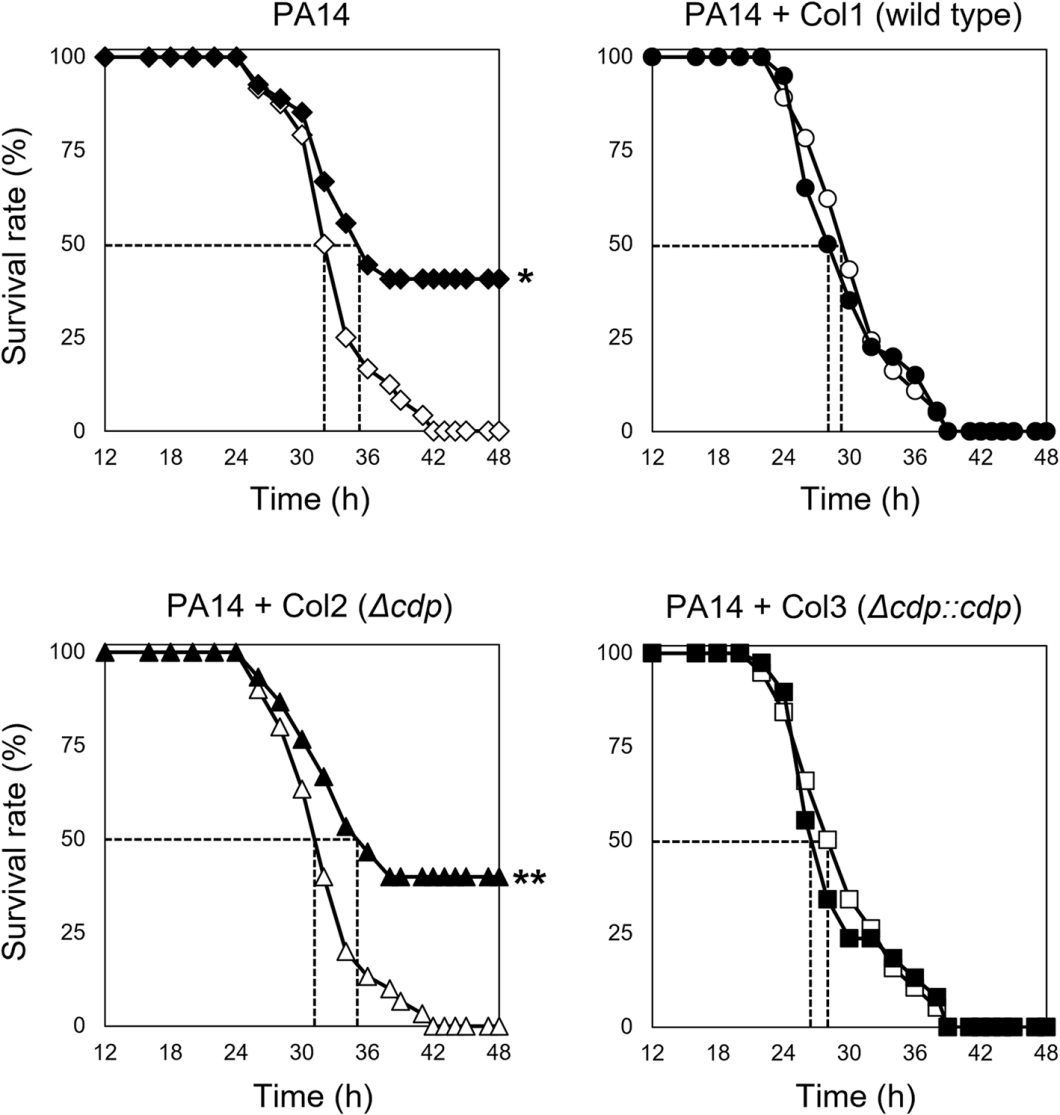
Antibacterial efficacy of colistin influenced by Cdp-producing *S. maltophilia* strains during *Drosophila* coinfection. Antibacterial efficacy of colistin was determined by the survival rate of *Drosophila* animals infected by *P. aeruginosa* PA14*. S. maltophilia* cells (strains Col1, Col2, and Col3) were coinfected with *P. aeruginosa* as described in the “Methods” section. The infected flies were fed with either 1 mg/mL colistin, and their survival rates were determined over time. The dotted lines represent the time required to reach 50% mortality. The statistical significance based on a log-rank test is indicated (*p = 0.0142; **p = 0.0058). Closed and open symbols indicate animals treated with and without colistin, respectively

Considering the complexity of actual polymicrobial infections in hosts, the aforementioned coculture results should be further verified by an appropriate animal infection model [13, 25, 31]. *Drosophila melanogaster* has been recognized as an animal model suitable for studying *P. aeruginosa* infection [32, 33]. *P. aeruginosa* strain PA14 was hired for fly infection due to its higher virulence than strain PAO1 [34]. The survival rates of *D. melanogaster* infected with strain PA14 or *S. maltophilia* Col1 were monitored for 48 h in the presence and absence of colistin. Colistin treatments did not affect the survival rate of flies. The monomicrobial infection with strain PA14 resulted in mortality of flies within 42 h (Fig. 4), while strain Col1 was avirulent to flies under the tested conditions. The colistin treatment for PA14 infection increased the survival rate of flies by up to 41%. However, when flies were coinfected with *S. maltophilia* strains carrying the *cdp* gene (strains Col1 and Col3), colistin treatment could not increase the survival rate. These results indicated that these *S. maltophilia* strains indeed provided colistin resistance for *P. aeruginosa*. In contrast, the coinfection with strain Col2 (*Δcdp*) resulted in a mortality rate as similar as the monomicrobial infection with PA14 only. In addition, in terms of the time required to reach a 50% mortality, the coinfection with *cdp*-carrying *S. maltophilia* strains killed the flies more slowly than the monomicrobial infection and the coinfection with strain Col2 devoid of the *cdp* gene (Fig. 4). Our results from the animal infection model approach also indicate that Cdp plays a pivotal role in collective resistance to colistin during polymicrobial infection.

### Evolutionary features of colistin-degrading enzymes among *S. maltophilia*

**Fig. 5:**
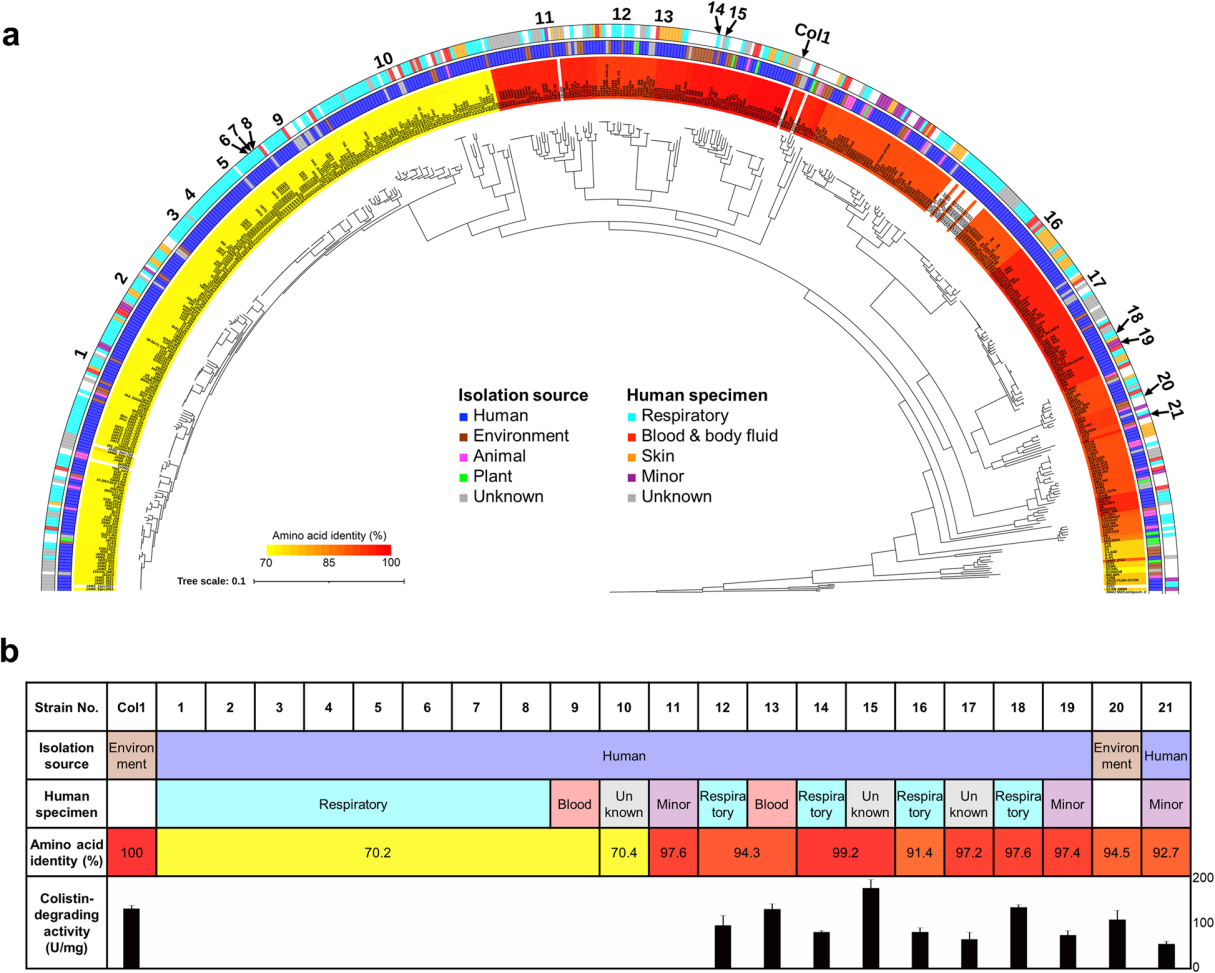
Phylogeny of *S. maltophilia* genomes associated with colistin-degrading proteases. **a** A genome-based phylogenetic tree was reconstructed by the maximum likelihood method using the concatenated alignments of 1073 core genes among *S. maltophilia* genomes. *S. rhizophila* was used as an outgroup. Shaded colors on strain names indicate amino acid sequence identity compared to that of the Cdp of strain Col1. Colors in the inner circle indicate the isolation source of strains: human (blue), environment (brown), animal (pink), plant (green), and unknown (gray). Colors in the outer circle indicate the different sources of human specimens: respiratory tract (cyan), blood and bodily fluid (red), skin (orange), minor specimen (purple), and unknown (gray). Strains tested for colistin-degrading activity are marked with numbers. **b** Characteristics of S. maltophilia strains tested for colistin-degrading activity

The phylogenomic tree constructed based on 1073 core gene sequences conserved in 551 *S. maltophilia* genomes is demonstrated with information on the source of isolation, including human specimen sources, in Fig. 5. The results showed that the *S. maltophilia* genomes were divided into several different genogroups (Fig. 5a), as suggested previously [35]. Most of *S. maltophilia* strains were isolated from humans (mainly from the respiratory tract) and others from animals, plants, and the environment. The majority of *S. maltophilia* genomes contained genes orthologous to the *cdp* gene, which were located in a region directly adjacent to the gene cluster of the type II secretion system (T2SS). These protease genes consisting of 154 unique amino acid sequences formed two major phylogenetic lineages with a few outliers (Additional file 1: Fig. S6). Interestingly, a large clade of *S. maltophilia* genomes possessed the distinctive lineage of the Cdp orthologs with relatively lower sequence identities (69.7–74.7%) to the Cdp of strain Col1 (shaded in yellow in Fig. 5a).

To understand the relationship between the genome phylogeny and the colistin-degrading activity of Cdp orthologs, 21 representative strains selected from two major lineages of Cdp orthologs were tested for the colistin-degrading activity (Fig. 5b). Notably, *S. maltophilia* strains producing proteases with relatively higher sequence identities (> 91.4%) to the Cdp of strain Col1 showed colistin-degrading activities (shaded in dark orange to red in Fig. 5a), with one exception. However, strains producing proteases with lower sequence identities (70.4–70.6%) to the Cdp of strain Col1 did not display the activity (Fig. 5b). Furthermore, all strains with the colistin-degrading activities provided protection to *P. aeruginosa* PAO1, which was revealed by the solid agar coculture experiment (Additional file 1: Fig. S6). It should be noted that among 10 strains displaying the positive colistin-degrading activities, nine strains were of human origin (Fig. 5b), suggesting that potential collective resistance providers are already prevalent in several *S. maltophilia* strains isolated from humans.

## Discussion

The emergence of MDR Gram-negative infections has required reconsideration of colistin as a treatment option [3]. Considering the rapid emergence and the subsequent dissemination of mobile colistin resistance mediated by *mcr-1* and its variants since its reintroduction to the clinic [6], the emergence of other resistance mechanisms is feasible. Furthermore, the chemical structure of colistin is vulnerable to degradation by proteolytic enzymes. Hence, these enzymes may be a novel resistance determinant [36]. Indeed, previous studies on colistin-degrading enzymes derived from *Bacillus licheniformis* and the colistin producer, *Paenibacillus polymyxa*, corroborate the presence of a colistin resistance mechanism mediated by such colistin-degrading enzymes [9, 10]. However, these proteases have not been clearly demonstrated to be an antimicrobial resistance determinant [11]. Considering that colistin is generally used for the treatment of Gram-negative pathogens, those taxa belonging to Gram-positive bacteria have not been considered urgent threats. In the present study, a colistin-degrading enzyme was first identified from the Gram-negative opportunistic pathogen *S. maltophilia*, which can confer multidrug resistance and is frequently isolated with other potent Gram-negative pathogens during respiratory tract infections, such as *P. aeruginosa* and *A*. *baumannii* [26, 37, 38]. We clearly demonstrated that Cdp inactivated the antimicrobial activity of colistin, thereby leading to the survival of bacteria carrying this gene. The expression of the protease and its contribution to bacterial survival was limited only to the late growth phase of strain Col1, which did not allow conventional AST methods to function, showing a highly significant MIC value. If the gene is present in a genetic context that enables an earlier and higher expression of the gene, it may lead to a significantly higher MIC value and be considered a more threatening resistance determinant.

Our discovery of colistin-degrading protease in the Gram-negative pathogen *S. maltophilia* has other important implications. A horizontal transfer of the protease-coding gene to more potent pathogens such as *P. aeruginosa* and *A. baumannii* may occur, because *S. maltophilia* is phylogenetically more related to these pathogens than *B. licheniformis* and *Paenibacillus polymyxa* and is often found together with these pathogens during respiratory tract infections [13, 25]. Until now, we have not observed any mobile traits of the protease genes in *S. maltophilia* genomes, but the genetic context must be thoroughly monitored for identifying their potential transferability in advance.

More importantly, the colistin-degrading enzyme can play a critical role in polymicrobial infection communities. In polymicrobial infections, strains carrying antibiotic-inactivating enzymes have been recognized as potential collective resistance providers, protecting concurrently infecting strains that may have been effectively killed by treated antibiotics [18, 22, 31, 39]. The carbapenem-resistant *A. baumannii* sheltered carbapenem-susceptible pathogens via an extracellular release of carbapenem-hydrolyzing class D β-lactamase [31]. Furthermore, enzymes capable of degrading or modifying several classes of antibiotics such as β-lactam, macrolide, tetracycline, and chloramphenicol have been shown to participate in indirect resistance [39]. Collective resistance was also observed via intracellular antibiotic deactivation, where chloramphenicol acetyltransferase-expressing pneumococci protected more susceptible strains [22]. The results of these studies clearly demonstrate that a microbial community involved in a polymicrobial infection can acquire collective resistance to antibiotics if a component of the community expresses antibiotic-inactivating enzymes. In the present study, results from coculture and animal infection experiments showed that *S. maltophilia* strains producing colistin-degrading enzymes could inactivate colistin and lower its concentration, providing protection to colistin-susceptible pathogens such as *P. aeruginosa* and *A. baumannii* for evading the antibiotic pressure. *S. maltophilia* is a member of bacterial communities of polymicrobial infection in CF patients [13, 38]. In the case of exacerbation of pulmonary infection attributed to the progressive invasion of *P. aeruginosa* in CF patients, concurrent infections with *S. maltophilia* strains expressing colistin-degrading enzymes may impede the successful treatment with the last-resort antibiotic colistin. A recent study revealed that *S. maltophilia* strains isolated from CF sputum could provide high levels of imipenem protection to otherwise sensitive *P. aeruginosa* via the chromosomally encoded metallo-β-lactamase [18]. These findings emphasize the importance of inter-species interaction in the ecological context that can alter antibiotic efficacy in bacterial communities of polymicrobial infection. However, despite the increasing prevalence of *S. maltophilia* coinfection with *P. aeruginosa* in CF patients, the impact of *S. maltophilia* on the treatment outcome of *P. aeruginosa* infections is poorly understood [25, 40, 41]. Notably, strains CV_2013 and CV_2003_STM1 that displayed colistin-degrading activities and colistin exposure protection in our study were actually isolated from the respiratory tract of CF patients. Therefore, the surveillance of colistin-inactivating protease should be carefully performed prior to colistin treatment for CF patients carrying *S. maltophilia* strains in the respiratory tracts.

Although *S. maltophilia* strain Col1 was isolated from the environment, a comparative genomics study indicated no significant phylogenetic and genomic differences between environmental and clinical isolates [42, 43]. Genomes of a number of *S. maltophilia* strains isolated from human specimens encoded proteases highly homologous to Cdp of strain Col1, and these proteases actually exhibited colistin-degrading activities, suggesting that *S. maltophilia* proteases, which are already prevalent in the clinical settings, should be regarded as novel resistance determinants. These proteases belong to the subfamily S08A of subtilisin protease to which the alkaline protease from *B. licheniformis* also belongs, but the Gram-positive enzyme showed a very low amino acid sequence identity (33.9%) with Cdp. Until now, colistin-degrading proteases were functionally identified only in a certain clade of *S. maltophilia* strains and some Gram-positive bacteria. A possibility can be raised that other proteolytic enzymes with colistin-degrading activity may have already evolved among a broader range of bacterial taxa [10, 44]. Notably, widely distributed bacterial peptidases mediated the hydrolytic cleavage of nonribosomal peptide antibiotics, including polymyxin, vancomycin, and teixobactin, which implicated broad-spectrum resistance and warned a potential risk if they are transferred to opportunistic pathogens [36]. In line with this notion, the prevalence of proteolytic enzymes with colistin-degrading activities in clinical settings may seriously affect the clinical efficacy of the last-resort drug for the treatment of patients infected by such protease-expressing bacteria. Therefore, exploring diverse colistin-degrading enzymes and their structural features would facilitate a better understanding of the proteolytic cleavage of colistin and provide more elaborate surveillance strategies in clinical settings.

In conclusion, our study suggests that the colistin-degrading protease should be recognized as an emerging colistin resistance determinant in the opportunistic pathogen *S. maltophilia*, which can also lead to collective resistance at the microbial community level during polymicrobial infections. Thus, we alarm the emergence of a novel colistin resistance mechanism as an imminent threat that should be under surveillance in clinical settings. This knowledge will also provide useful information for successful clinical outcomes during the treatment of complex polymicrobial infections, particularly including *S. maltophilia* and other colistin-susceptible Gram-negative pathogens.

## Methods

### Isolation of a colistin-degrading bacterium

A colistin-degrading bacterial strain, Col1, was isolated from urban soil in South Korea. The soil sample was incubated at 30 °C for 2 days in R2A broth (MB cell, Korea), followed by the addition of colistin at a concentration of 100 mg/L (Sigma-Aldrich, USA). After repeated subcultures, a pure culture was obtained. The isolated bacteria were routinely cultivated in the R2A medium at 37 °C.

### Genome sequencing

The genomic DNA of strain Col1 was extracted using DNeasy Blood & Tissue Kit (Qiagen, USA) according to the manufacturer’s instructions. Whole-genome sequencing and assembly were performed using the PacBio RS II (Pacific Biosciences, USA) platform at CJ Bioscience (Seoul, Korea). Functional annotation was performed using the SEED and COG databases [45, 46]. The genome sequence has been deposited in the NCBI GenBank database with the accession number CP077679.

### Colistin inactivation assay

The culture supernatant was obtained by centrifugation at 13,000 × *g* for 10 min and filtration (0.2 μm). To assess the colistin-inactivating activity, the supernatant was reacted with 500 mg/L colistin sulfate in 50 mM Tris–HCl (pH 8.5) at 50 °C. Residual antimicrobial activity from the reaction mixture was tested using the disk diffusion assay against colistin-susceptible *E. coli* DH5α according to the CLSI guidelines [47].

### HPLC and LC–MS/MS analyses for colistin and its metabolites

Cleavage of colistin and production of the concomitant metabolites in the aforementioned reaction were analyzed by high-performance liquid chromatography (HPLC) using a Kinetex C-18 column (Phenomenex, USA) and an Alltech 3300 Evaporative Light Scattering Detector (ELSD) (BUCHI, Switzerland). The mobile phase comprised a gradient of 24 to 29% acetonitrile (J.T. Baker) and 0.021 to 0.024% trifluoroacetic acid (Sigma-Aldrich) with a flow rate of 1.0 mL/min for 10 min. Detection was performed at 60 °C and 1.5 mL/min nitrogen gas flow by ELSD. A linear ion trap mass spectrometer (LTQ-Velos, Thermo Scientific, USA) with a nano sprayer coupled to the Accela HPLC system (Thermo Scientific) was used for LC–MS/MS analysis [48]. The Xcalibur software v. 2.1 (Thermo Scientific) was used for tandem mass spectral data analysis. A chemically synthesized authentic compound of the cyclic peptide moiety of colistin (Peptron, Korea) was used to confirm the chemical structure of the metabolite using MS/MS fingerprinting.

### Purification and identification of colistin-degrading enzyme

*S. maltophilia* Col1 was cultured in 500 mL of R2A broth at 37 °C for 12 h. The culture supernatant was concentrated by ultrafiltration using a 5-kDa molecular weight cutoff membrane (Merck Millipore). The concentrated solution was loaded onto a column packed with Q Sepharose fast flow (GE Healthcare) equilibrated with buffer A (50 mM Tris–HCl, pH 7.4) containing 1 mM dithiothreitol (DTT). Proteins were eluted by a linear gradient of 0 to 0.6 M NaCl in the same buffer at a flow rate of 5 mL/min. The fractions showing colistin-degrading activity were collected and concentrated. The active fractions were further separated by a gel filtration chromatography (Superdex, GE Healthcare) using buffer B (50 mM Tris–HCl, pH 7.4) containing 1 mM DTT and 0.1 mM NaCl at a flow rate of 0.5 mL/min. The active fractions were collected and concentrated. The concentrate was applied to a Mono-Q 5/50 GL column (GE Healthcare) equilibrated with buffer A, and proteins were eluted using a linear gradient of 0 to 0.5 M NaCl at a flow rate of 1 mL/min. One enzyme unit is defined as the amount of enzyme required to consume 1 nmole colistin B per minute at 50 °C. The purified protein was visualized by sodium dodecyl sulfate–polyacrylamide gel electrophoresis (SDS-PAGE) analysis. For protein identification, protein bands were excised and digested with trypsin. The tryptic peptides were analyzed by LC–MS/MS as described previously [49]. The peptide spectra were searched against the genome sequence of strain Col1 using the SEQUEST algorithm implemented in the Proteome Discoverer 1.3 software (Thermo Scientific).

### Construction of knock-out and complementation mutants

A knock-out mutant (*Δcdp*) of *S. maltophilia* was constructed using the allelic exchange method [50]. The used primers were summarized in Additional file 1: Table S1. About 1000-bp fragments of upstream and downstream regions of a target gene were obtained by PCR amplification. The generated fragments were fused by overlap extension PCR and a 1943-bp fragment consisting of the upstream and downstream flanking sequences without the target gene was amplified by PCR using a nested primer pair. The fragment was cloned into a pEX18Tc vector, and the vector construct was introduced to strain Col1 by electroporation. To obtain a target-gene deleted mutant, single and double homologous recombinants were selected sequentially using tetracycline and sucrose as selection markers, respectively. The deletion of the target gene was confirmed by PCR. For the complementation mutant (*Δcdp::cdp*), coding and promoter regions of the target gene were inserted at the downstream of lac promoter in the pBBR1MCS-3 vector. The resulting plasmid was introduced to the mutant, and a tetracycline-resistant transformant was selected as a complemented strain. The introduction of the vector was further confirmed by PCR amplification and sequencing. These mutants were employed for growth inhibition experiments and colistin exposure protection assays.

### Growth inhibition by colistin

Growth of wild-type and mutant strains and colistin-cleavage activity of the culture supernatants were monitored every 2 h. For growth, viable cell numbers were monitored for up to 4 h after colistin (128 mg/L) was added to bacterial cell cultures (10^9^ CFU/mL) of *S. maltophilia* strains. Residual amounts of colistin were analyzed by HPLC.

### Colistin exposure protection assays

Survival of *P. aeruginosa* strain PAO1 mediated by *S. maltophilia* strain Col1 or its mutants was analyzed by performing the MIC test and planktonic and solid-agar coculture experiments. The MIC test of strain PAO1 was performed when the culture supernatant of strain Col1 or its mutants was added to the R2A medium [51]. To demonstrate the impact of protease-producing bacteria on colistin-susceptible bacteria, we developed a solid agar coculture assay described previously by Hernandez-Valdes et al. with modifications [52]. Strain Col1 or its mutants (10^5^ CFU/mL) were inoculated as lawn culture using a cotton swab, and cells of strain PAO1 (10^5^ CFU) were spotted at a distance of 1 cm to the lawn in the presence and absence of colistin (10 mg/L). For the planktonic coculture experiment, each of colistin-susceptible strains PAO1 (10^8^ CFU/mL) and *Acinetobacter baumannii* ATCC 17,978 (10^8^ CFU/mL) was mixed with strain Col1 or its mutants (10^9^ CFU/mL) in the R2A broth. Colistin was added to the mixed cultures at a concentration of 32 mg/L, which is effective only for the susceptible strains. Viable cell numbers of both strains in the cocultures were monitored for up to 4 h after the colistin spike.

### Evaluation of antibacterial efficacy against bacterial infection

*Drosophila* systemic infection was performed as previously described [33]. Briefly, *Drosophila melanogaster* strain Oregon R was grown and maintained at 25 °C using the corn meal-dextrose medium [0.93% agar, 6.24% dry yeast, 4.08% corn meal, 8.62% dextrose, 0.1% methyl paraben, and 0.45% (v/v) propionic acid]. For systemic infection, 4- to 5-day-old adult female flies were infected by pricking at the dorsal thorax with a 0.4 mm needle (Ernest F. Fullam, Inc.). The needle was dipped into a PBS-diluted bacterial suspension containing *P. aeruginosa* PA14 (10^7^ CFU/ml) and/or *S. maltophilia* strains (10^7^ CFU/ml). For colistin treatment, the flies were fed with 1 mg/ml colistin. Survival rates of infected flies were monitored for up to 48 h after infection. Flies that died within 12 h were excluded in mortality determination. Mortality assay was repeated at least three times.

### Phylogenomic and comparative genomic analyses

The genome assembly data of 550 *S. maltophilia* strains with high-quality sequences and *S. rhizophila* DSM 14,405 (as an outgroup) obtained from the NCBI RefSeq database were used for comparison with the genome of strain Col1. A phylogenomic tree of 551 genomes was reconstructed using 1073 core genes conserved in those genomes. Maximum likelihood phylogenetic analysis was performed using FastTree [53], and Interactive Tree of Life (iTOL) v4 was used to visualize the tree [54]. Isolation source information was collected from the BioSample database of NCBI.

## Supplementary Information

**Additional file 1:** Supplementary information file. **Fig. S1.** MS and MS/MS spectra of authentic compounds of colistin. **Fig. S2. **MS and MS/MS spectra of colistin metabolites. **Fig. S3. **Comparison of MS and MS/MS fragmentation spectra between metabolite 3 produced by strain Col1 (a) and the chemically synthesized compound of cyclic peptide (b). **Fig. S4. **Growth and colistin-degrading activity of *S. maltophilia *strains Col1 (a), Col2 (b), and Col3 (c). **Fig. S5. **Colistin exposure protection to *A. baumannii* provided by Cdp-producing *S. maltophilia* strains. **Fig. S6. **Phylogeny of Cdp and its orthologous proteins representing colistin-degrading activity and colistin exposure protection. **Table S1. **Oligonucleotide primers used in this study.

## Data Availability

The genome sequence of strain Col1 has been deposited in the NCBI GenBank database under the accession number CP077679.
