# Supplementary material for: Colistin-degrading proteases confer collective resistance to microbial communities during polymicrobial infections
Source: Microbiome. 2022 Aug 19;10:129. doi: 10.1186/s40168-022-01315-x (PMC9389796; doi:10.1186/s40168-022-01315-x)
Supplement: Supplementary file 2 — Additional file 1: Supplementary information file. Fig. S1. MS and MS/MS spectra of authentic compounds of colistin. Fig. S2. MS and MS/MS spectra of colistin metabolites. Fig. S3. Comparison of MS and MS/MS fragmentation spectra between metabolite 3 produced by strain Col1 (a) and the chemically synthesized compound of cyclic peptide (b). Fig. S4. Growth and colistin-degrading activity of S. maltophilia strains Col1 (a), Col2 (b), and Col3 (c). Fig. S5. Colistin exposure protection to A. baumannii provided by Cdp-producing S. maltophilia strains. Fig. S6. Phylogeny of Cdp and its orthologous proteins representing colistin-degrading activity and colistin exposure protection. Table S1. Oligonucleotide primers used in this study. [file 40168_2022_1315_MOESM1_ESM.docx]

**Additional file 1 for**

**Colistin-degrading proteases confer collective resistance in polymicrobial infection communities**

Do-Hoon Lee^1†^, Ju-Hee Cha^1†^, Dae-Wi Kim^1,2†^, Kihyun Lee^1^, Yong-Seok Kim^1^, Hyo-Young Oh^3^, You-Hee Cho^3^, and Chang-Jun Cha^1*^

^*^Correspondence: cjcha@cau.ac.kr

**This file includes:**

**Fig. S1** MS and MS/MS spectra of authentic compounds of colistin.

**Fig. S2** MS and MS/MS spectra of colistin metabolites.

**Fig. S3** Comparison of MS and MS/MS fragmentation spectra between metabolite 3 produced by strain Col1 (a) and the chemically synthesized compound of cyclic peptide (b).

**Fig. S4** Growth and colistin-degrading activity of *S. maltophilia* strains Col1 (a), Col2 (b), and Col3 (c).

**Fig. S5** Colistin exposure protection to *A. baumannii* provided by Cdp-producing *S. maltophilia* strains.

**Fig. S6** Phylogeny of Cdp and its orthologous proteins representing colistin-degrading activity and colistin exposure protection.

**Table S1** Oligonucleotide primers used in this study.

**
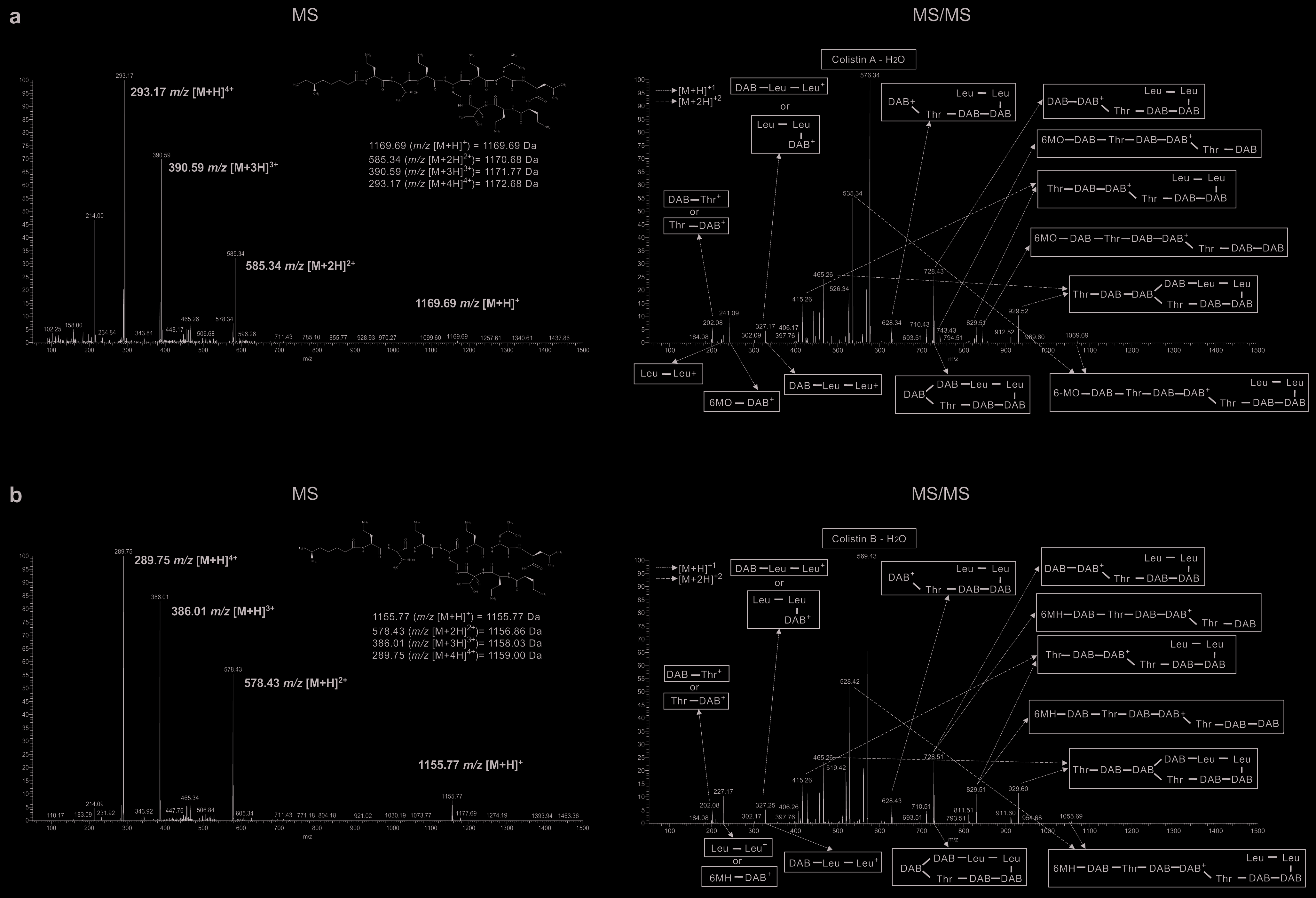

Fig. S1** MS and MS/MS spectra of authentic compounds of colistin. **a** colistin A. **b** colistin B.





**Fig. S2** MS and MS/MS spectra of colistin metabolites. **a** metabolite 1. **b** metabolite 2. **c** metabolite 3.


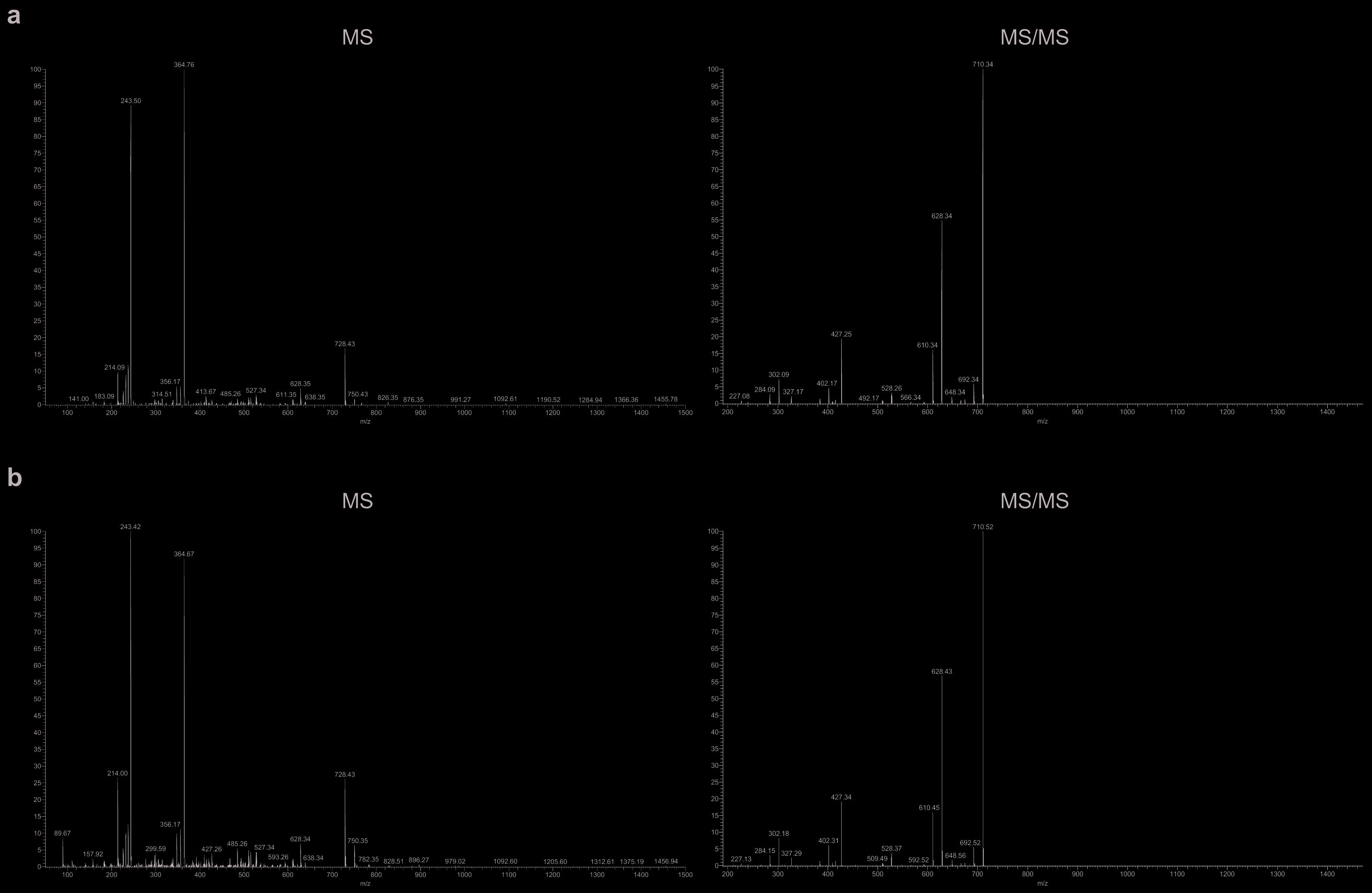

**Fig. S3** Comparison of MS and MS/MS fragmentation spectra between metabolite 3 produced by strain Col1 (a) and the chemically synthesized compound of cyclic peptide (b).

**
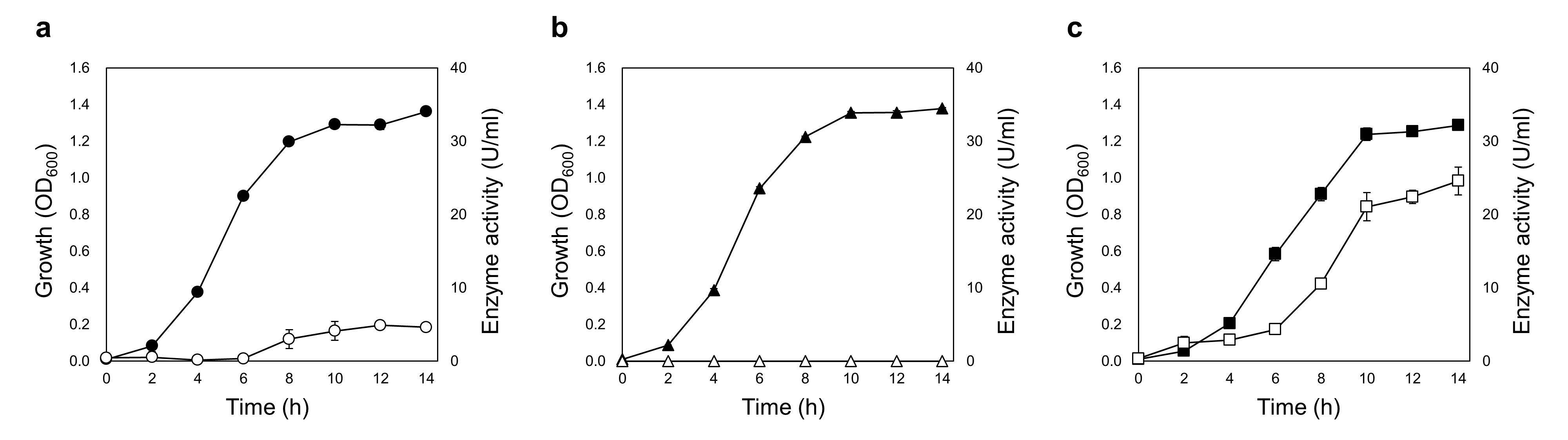
**

**Fig. S4** Growth and colistin-degrading activity of *S. maltophilia* strains Col1 (a), Col2 (b), and Col3 (c). Closed and open symbols indicate bacterial growth and colistin-degrading activity, respectively.


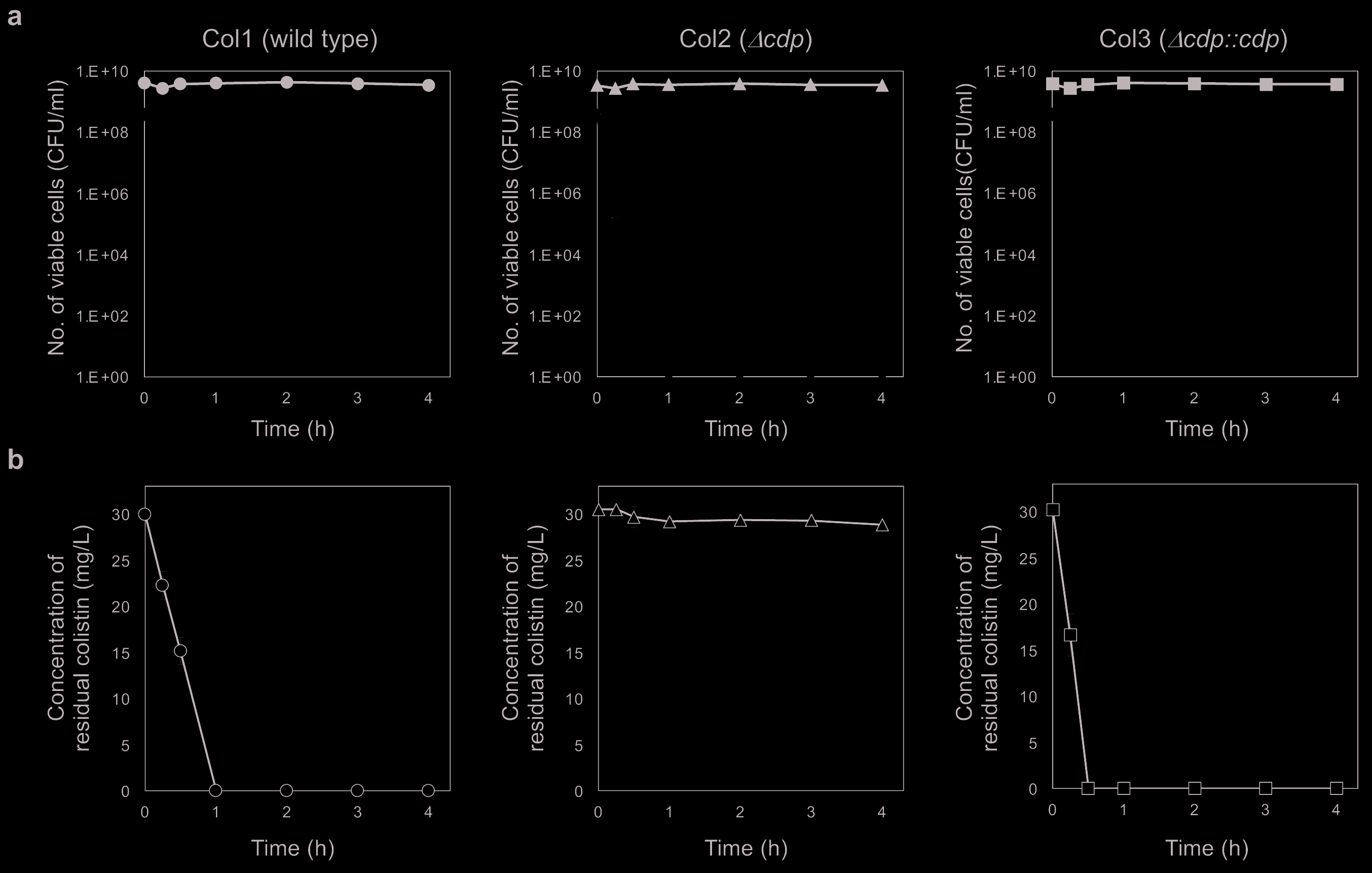


**Fig. S5** Colistin exposure protection to *A. baumannii* provided by Cdp-producing *S. maltophilia* strains. **a** Changes in viable cell numbers of *A. baumannii* strain ATCC 17978 (red) and *S. maltophilia* strains (black). **b** Concentration of residual colistin in the co-cultures after colistin spike (32 mg/L).


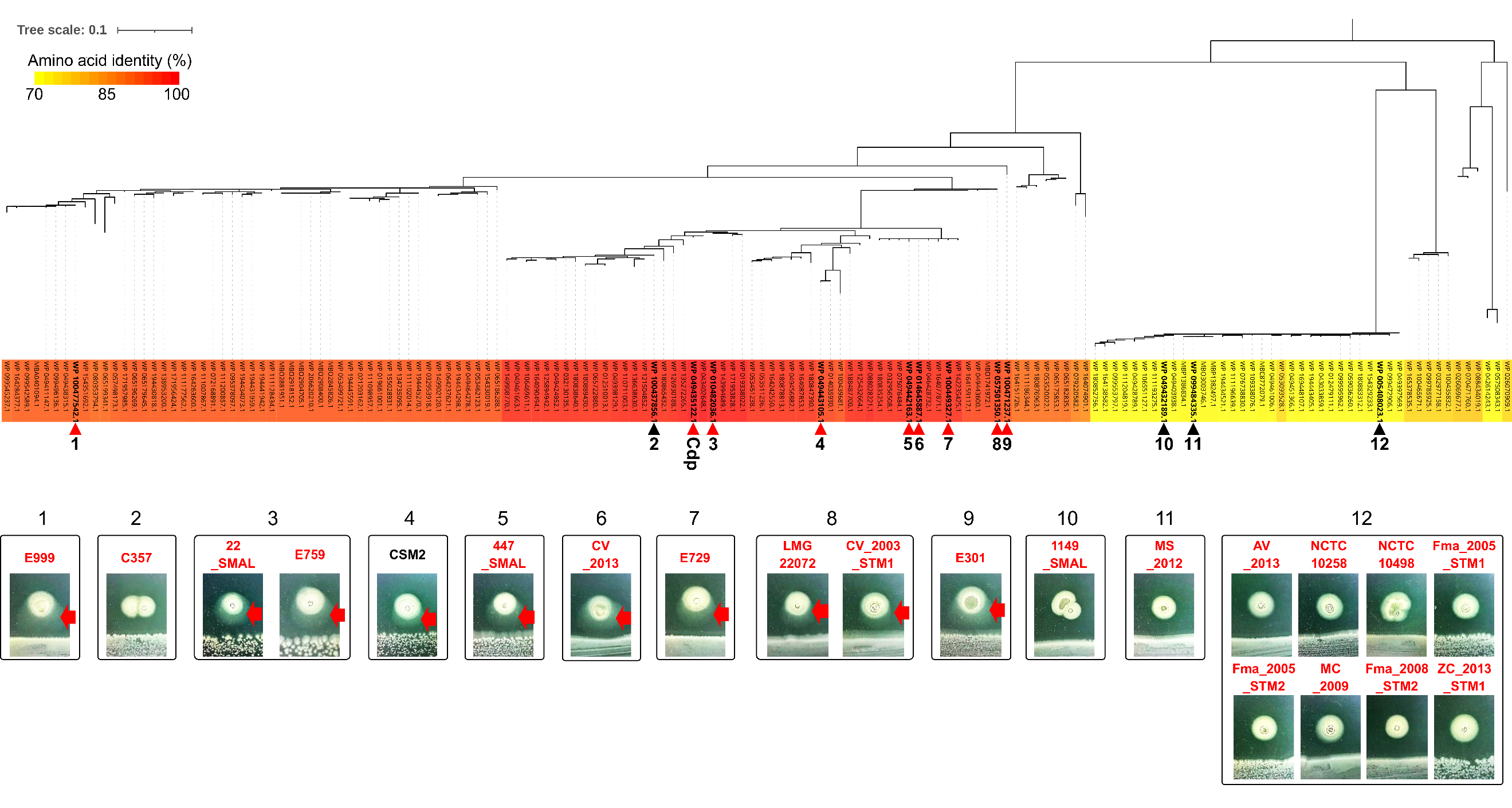


**Fig. S6** Phylogeny of Cdp and its orthologous proteins representing colistin-degrading activity and colistin exposure protection. Colors were shaded on the accession numbers of 154 unique amino acid sequences of proteases derived from 551 *S. maltophilia* genomes according to the sequence identity to Cdp of strain Col1. Twenty-one *S. maltophilia* strains selected from two major lineages of proteases were tested for colistin-degrading activity. Red and black triangles indicate positive and negative activities, respectively. Numbers indicate 12 unique protease sequences from 21 *S. maltophilia* strains. Red arrows indicate the spreading growth of *P. aeruginosa* strain PAO1 toward *S. maltophilia* strains. Strain names written in red indicate isolates from human specimen.

**Table S1** Oligonucleotide primers used in this study.

| Primer name | Sequence (5′-3′) | Amplicon size (bp) | Usage |
| --- | --- | --- | --- |
| 00541_up_F | ATCCAGAGCTCCACCTACAGCAACGTCGG | 1,027 | To amplify the upstream region of target gene (00541) |
| 00541_up_R | TTCAACTACGACAGTTCCAGCTTACACGTC GAATTCCTCTTCTGGGTCATGGAT |  |  |
| 00541_down_F | ACCCAGAAGAGGAATTCGACGTGTAAG CTGAACTGTCGTAGTTGGAATGGCCGA | 1,037 | To amplify the downstream region of target gene (00541) |
| 00541_down_R | ATCACCAAGCTTTCGCCGTGCGCGGTCG |  |  |
| 00541_nest_F | TCGGCGCGCTGGACCGCAAGGTCAGC | 1,943 | To amplify target gene (00541)-deleted fragment to clone into pEX18Tc vector |
| 00541_nest_R | AGGTCCAGTCTAGAGCCCTGCACGCGTAGC |  |  |
| Colony_F | AGGCCGTCGCCGACAGCTT | 2,792 | To confirm target gene (00541)-deletion |
| Colony_R | ACAGCCGTACGCCCTGTTCA |  |  |
| 00541_wPro_F | ACCAGTGGGCCCGGTATCCGCACC | 2,322 | To amplify target gene (00541) and 334 bp upstream region of the gene to clone into pBBR1MCS_3 vector To confirm the introduction of target gene (00541) in the deletion mutant |
| 00541_wPro_R | CCTGTCAATGAGCTCGACTTTAACAG |  |  |
